# Combining ultrasonic guided wave and low-frequency electromagnetic technology for defect detection in high-temperature Cr–Ni alloy furnace tubes

**DOI:** 10.1038/s41598-023-45627-w

**Published:** 2023-10-30

**Authors:** Chenyang Du, Xiaowei Li, Chang Liu, Ce Song, Jun Yuan, Yanchao Xin

**Affiliations:** 1 China Special Equipment Inspection and Research Institute, Beijing, 100029 China; 2 Technology Innovation Center of Risk Prevention and Control of Refining and Chemical Equipment for State Market Regulation, Beijing, 100029 China

**Keywords:** Energy science and technology, Engineering, Materials science

## Abstract

Cracking furnaces, operating under high temperatures and in a hydrocarbon medium, subject their tubes to complex stresses such as internal pressure, self-weight, fatigue, and thermal shock during start-up and shutdown. As a result, these furnace tubes frequently experience failures characterized by cracks and corrosion perforation. The high-temperature environment, constantly evolving structure of the tubes, and the close arrangement of the cracks within the tube box hinder detecting the cracks using conventional single-detection methods is challenging. This paper breaks through the limitations of the traditional single detection method and studies the effectiveness of the combination of ultrasonic-guided wave and low-frequency electromagnetic detection methods. The experiment was carried out by deliberately making cracks and thinning defects caused by corrosion on the cracking furnace tube of Cr35Ni45Nb after two years of service. The experimental results show that the ultrasonic guided wave detection technology can quickly detect the defects running through the whole furnace tube and effectively identify the manufacturing defects. On the other hand, low-frequency electromagnetic detection makes it possible to scan suspicious local defects and make qualitative and quantitative analyses of defect signals. The combination of ultrasonic guided wave and low-frequency electromagnetic detection can realize the rapid location and comprehensive qualitative and quantitative analysis of furnace tube defects, thus making up for the defects missed detection caused by the lack of effectiveness of single detection and the resulting safety problems. The research results have great popularization value in practical engineering applications.

## Introduction

The ethylene plant is prominent in the chemical industry, utilizing naphtha as a raw material for ethylene production through cracking reactions. Operating at high temperatures (e.g., 1100 °C) for extended periods, the furnace tube endures complex stresses, including internal pressure, self-weight, fatigue, and thermal shock resulting from start-up and shutdown processes. Consequently, occasional failures occur in cracking furnace tubes, where the medium reacts^1,2^. These failures occur for multiple reasons^3–5^ associated with various factors. Cracking, perforation, and bending are forms of ultimate physical failure^6–8^. During prolonged high-temperature service, the microstructure and properties of the furnace tube undergo continuous changes. The dendritic structure characteristics in the examined furnace tubes persisted after 2 years of service, while the interdendritic eutectic carbide transformed. The original white bone carbide NbC evolved into a block or strip, and the strip of Cr-rich carbide between dendrites grew, tending to form a network.

Existing nondestructive testing methods for detecting defects in cracking furnace tubes mainly include ultrasonic testing, carburizing testing, acoustic emission testing, infrared thermal imaging testing, and magnetic testing. Ultrasonic and acoustic emission detection primarily determine furnace tube defects and damages through time difference positioning. They assess the degree of deterioration based on acoustic emission or Backhausen noise signals, offering convenient detection of defects such as cracks^9,10^. However, these methods are susceptible to environmental interference, leading to potential errors. Infrared thermal imaging detection primarily focuses on operational cracking furnace detection, enabling real-time monitoring of the furnace tube temperature^11,12^. However, this method is primarily employed for furnace temperature monitoring and does not detect defects such as cracking and corrosion in the furnace tube. Magnetic detection utilizes the mechanism where carburization or oxidation of the furnace tube’s material results in a shift from paramagnetic to ferromagnetic properties^13^. Magnetic detection is performed by leveraging the relationship between changes in tube structure and magnetic properties^14–18^. However, these methods are primarily used to assess the degree of carburization damage in furnace tubes and are still in the experimental research stage. Given the structural characteristics of the cracking furnace, such as its location in the tube box, narrow space, and elongated length, ultrasonic guided waves offer advantages like an extended detection range and rapid detection speed, facilitating swift defect localization. Furthermore, low-frequency electromagnetic detection technology allows for localized scanning of defects and suspicious areas. After instrument calibration on the calibration tube, defects and suspicious signals can be qualitatively and quantitatively analyzed. Therefore, the present study investigated the combined detection of cracks and corrosion defects in cracking furnace tubes using ultrasonic guided waves and low-frequency electromagnetism to address the challenges associated with detecting defects in cracking furnace tubes.

In this study, we selected two cracking furnace tubes that have been in service for 2 years as experimental specimens. Artificial defects, including cracks and corrosion, were deliberately created on the tubes. We performed experiments employing ultrasonic guided wave detection and low-frequency electromagnetic detection on the specimens, followed by statistical analysis of the detection data. Furthermore, we explored the application of ultrasonic guided wave detection and low-frequency electromagnetic detection technology for assessing cracking and corrosion in cracking furnace tubes. The findings of this study provide an empirical and scientific basis for their broader adoption and implementation.

## Microstructure evolution of cracking furnace tube in high-temperature service

The microstructure of furnace tube materials undergoes significant changes when subjected to prolonged exposure to high temperatures. While numerous studies have focused on the evolution of microstructure and performance degradation in Cr25Ni20 and Cr25Ni35 alloy types, research on Cr35Ni45-type cracking furnace tubes during service is limited. This study focused on the microstructure evolution of Cr35Ni45-type furnace tube materials with varying service times (not in service and 2 years in service) by comparing them with the microstructure of unused furnace tube materials. The aim was to gain insights into the evolutionary characteristics of the microstructure after 2 years of operation.

### Experimental materials and methods

The experimental materials included furnace tubes in service for 2 years and new, unused tubes. The actual operating temperature of the furnace was 1080 ℃, and the material of the furnace tubes was Cr35Ni45Nb, with dimensions of Ф 120 × 10 × 2000 mm. The furnace tubes were manufactured using centrifugal casting. The chemical composition of the original as-cast furnace tube material is presented in Table 1.

**Table 1:** Chemical composition of the as-cast furnace tubes.

Element	C	Nb	Cr	Ni	Ti	Si	Fe
Percentage /(wt,%)	0.5	1.0	35.44	43.57	0.01	1.6	17.88

Two furnace tubes were selected for the experiment: one that had not been in service (labeled as specimen “a”) and one with a service life of 2 years (labeled as specimen “b”). Arc-shaped block specimens measuring 10 × 15 × 7 mm were cut from these tubes. The specimens were polished using sanding techniques and then underwent electrolytic etching at a voltage of 5 V for 5 s using an electrolytic solution composed of 150 ml H_3_PO_4_, 10 ml H_2_SO_4_, and 15 g CrO_3_. The histomorphological features and phase composition of the specimens were observed using an optical microscope (9XB-PC type), a scanning electron microscope (JSM-6510A), and an electron microprobe.

Furthermore, a 20 mm section was cut from each furnace tube to remove the oxide layer on the inner and outer walls. The extracted samples were subjected to extraction at 5 V DC for 15 h using a composition of 10% HCl + 90% CH_3_OH (v/v). The extracted products were then analyzed using X-ray diffraction (XRD) with a Rigaku D/MAX-RB diffractometer.

### Experimental results

**Figure 1:**
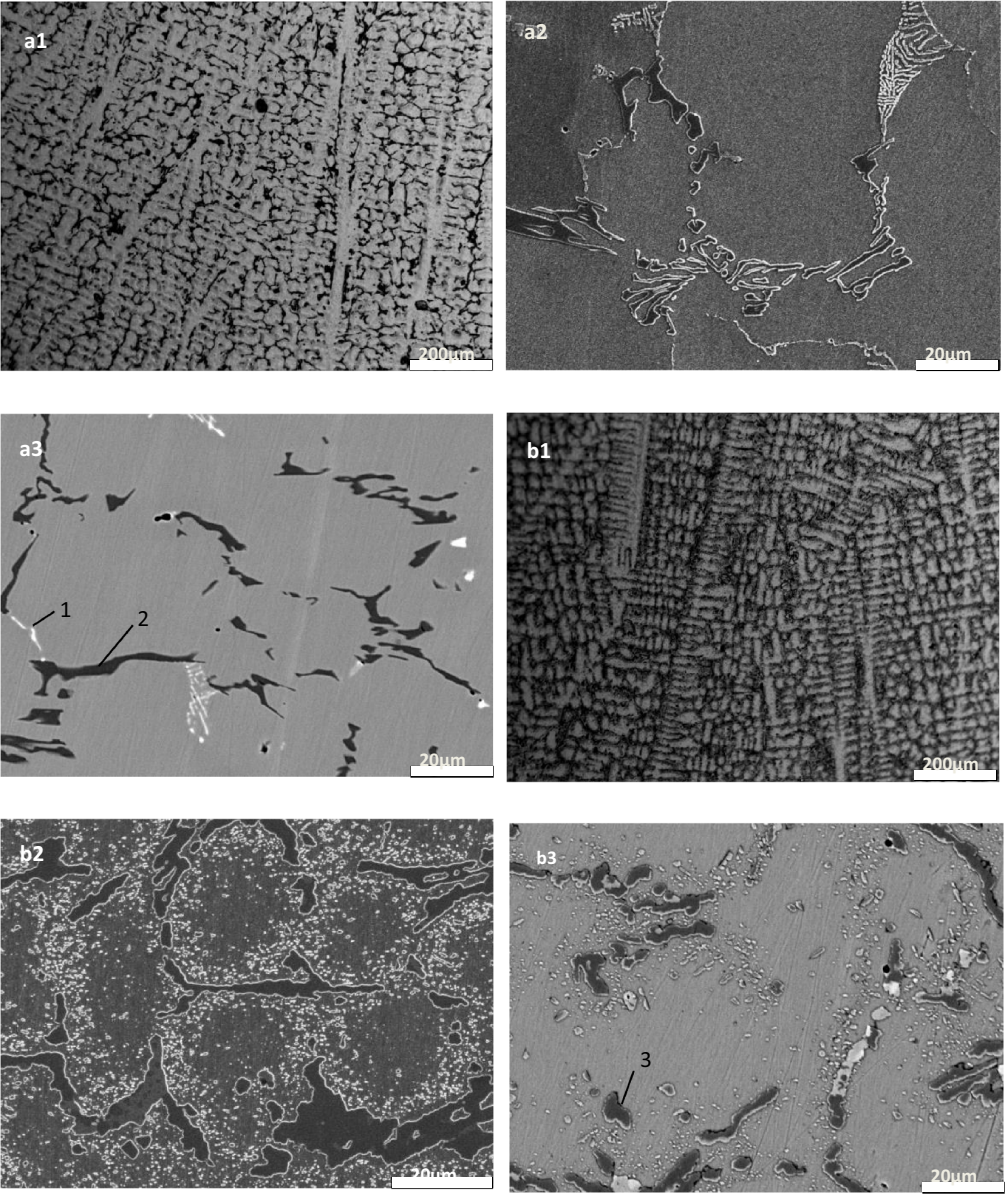
Microstructure characteristics of furnace tubes under different service time. (**a**) Not in service; (**b**) 2 years’ service.

**Figure 2:**
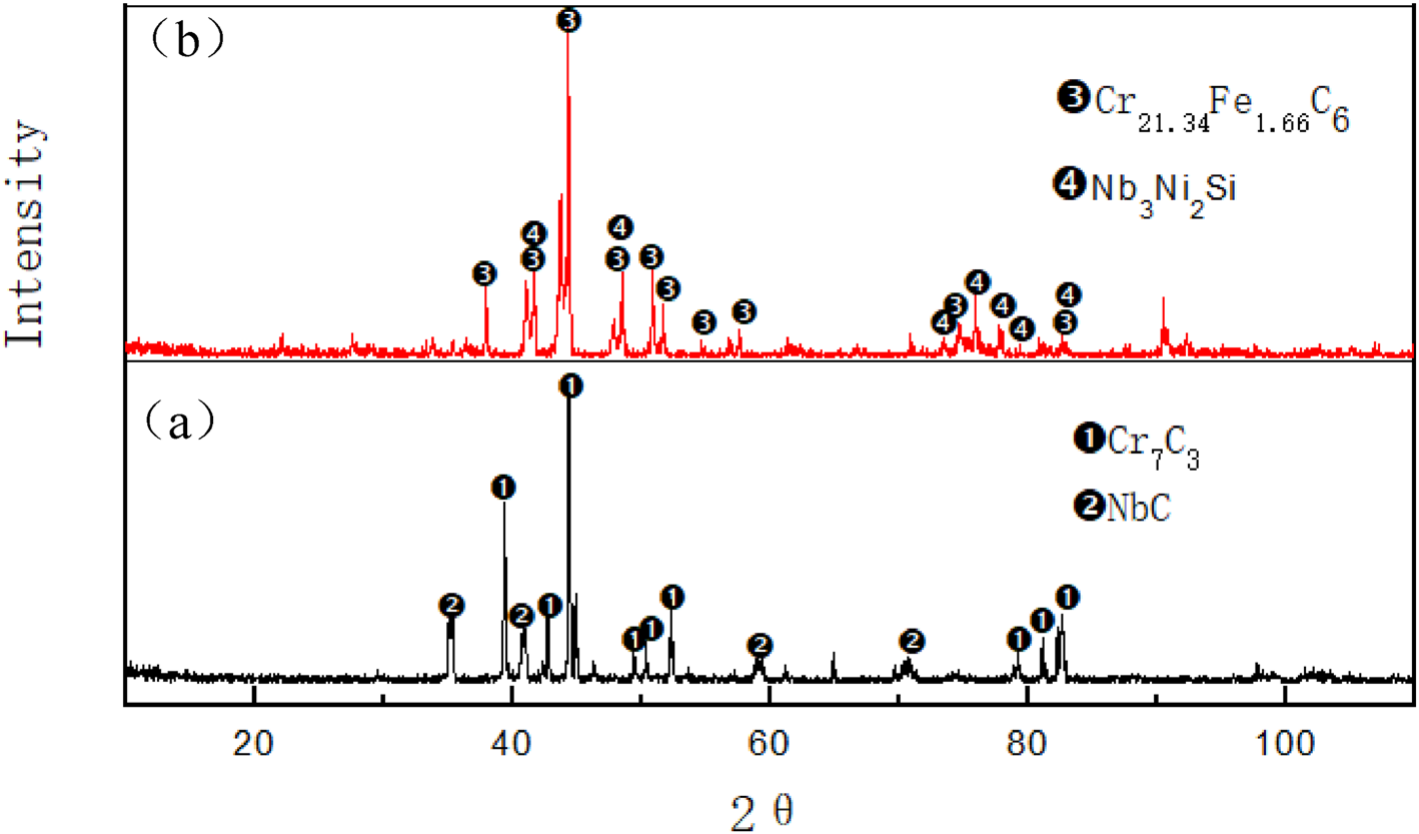
Evolution of carbide structure under different service conditions. (**a**) Not in service; (**b**) 2 years’ service.

The microstructural characteristics of the furnace tubes under different service times were examined, and the results are presented in Fig. 1. When observed under an optical microscope in the unused state (Fig. 1a), the furnace tube exhibited a typical coarse dendritic structure. Further analysis using a scanning electron microscope revealed two types of dendrites in the original as-cast furnace tube: one with a bright white fishbone structure and the other with a gray-black strip structure, as depicted in the secondary electron image (Fig. 1a2). The backscattered electron image (Fig. 1a3) confirmed that the black and white phases in Fig. 1a2 corresponded to two different precipitated phases. Quantitative composition analysis using an electron probe (Table 2) and XRD analysis confirmed (Fig. 2a) that the white fishbone structure (Fig. 1a3) corresponded to the eutectic NbC, while the gray-black strip structure (Fig. 1a3) corresponded to Cr-rich M_7_C_3_ carbide.

**Figure 3:**
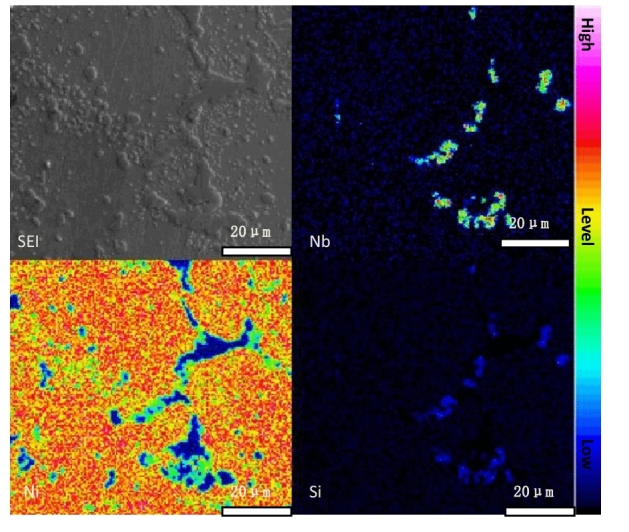
Scanning spectrum of the electron probe in a furnace tube in service for 2 years.

**Table 2:** Electron probe fixed-point analysis (WDS) results.

Element/atom%		C	Si	Cr	Ni	Fe	Nb	Phase
Position	1	38.01	0.79	8.17	7.32	2.24	43.46	NbC
	2	34.48	0.06	62.45	0.88	1.67	0.46	M_7_C_3_
	3	21.26	0.00	69.82	3.53	5.33	0.05	M_23_C_6_

As illustrated in Fig. 1b1, even after 2 years of service, the furnace tube retained its dendritic structure characteristics, but the interdendritic eutectic carbide underwent some changes. In particular, the original white bone carbide NbC had transformed into block or strip-shaped structures, and the elongated Cr-rich carbide between dendrites had significantly grown and started to form a network. Scanning spectrum analysis of the electron probe (Fig. 3) revealed that NbC had transformed into niobium-nickel silicide during service, with visible element distribution within the silicide. XRD results in Fig. 2b indicated that this niobium-nickel silicide was Nb_3_Ni_2_Si, which appeared grayish-white in the backscattered electron image. The transformation into Nb3Ni2Si after 2 years of service was incomplete, resulting in the coexistence of black, grayish-white, and bright-white structures in Fig. 1b3. XRD analysis further confirmed that the Cr-rich M_7_C_3_ carbide had completely transformed into M_23_C_6_ carbide during service. Additionally, many granular M_23_C_6_ secondary carbides had precipitated within the crystals and tended to aggregate between dendrites.

## Non-destructive testing experimental specimens, instruments, and methods

### Experimental specimens

The experimental specimens used in this study were cracking furnace tubes that had been in service for 2 years. These tubes were taken from the same cracking furnace. Detailed information about the experimental materials and their specifications can be found in Table 3.

**Table 3:** Experimental materials and specifications.

Specimen name	Defect type	Material	Furnace tube specifications (mm)	Furnace tube status
Simulation of cracked defective stovepipes	Crackle	Cr35Ni45Nb	Ф120 × 10 × 2000	Served 2 years
Simulation of corrosion defective furnace tubes	Corrosive	Cr35Ni45Nb	Ф120 × 10 × 2000	Served 2 years

Artificial simulated defects were introduced into these specimens through machining. Ultrasonic guided wave detection and low-frequency electromagnetic detection tests were conducted to evaluate the effectiveness of these methods in detecting the simulated defects.

### Test piece defect setting

**Figure 4:**
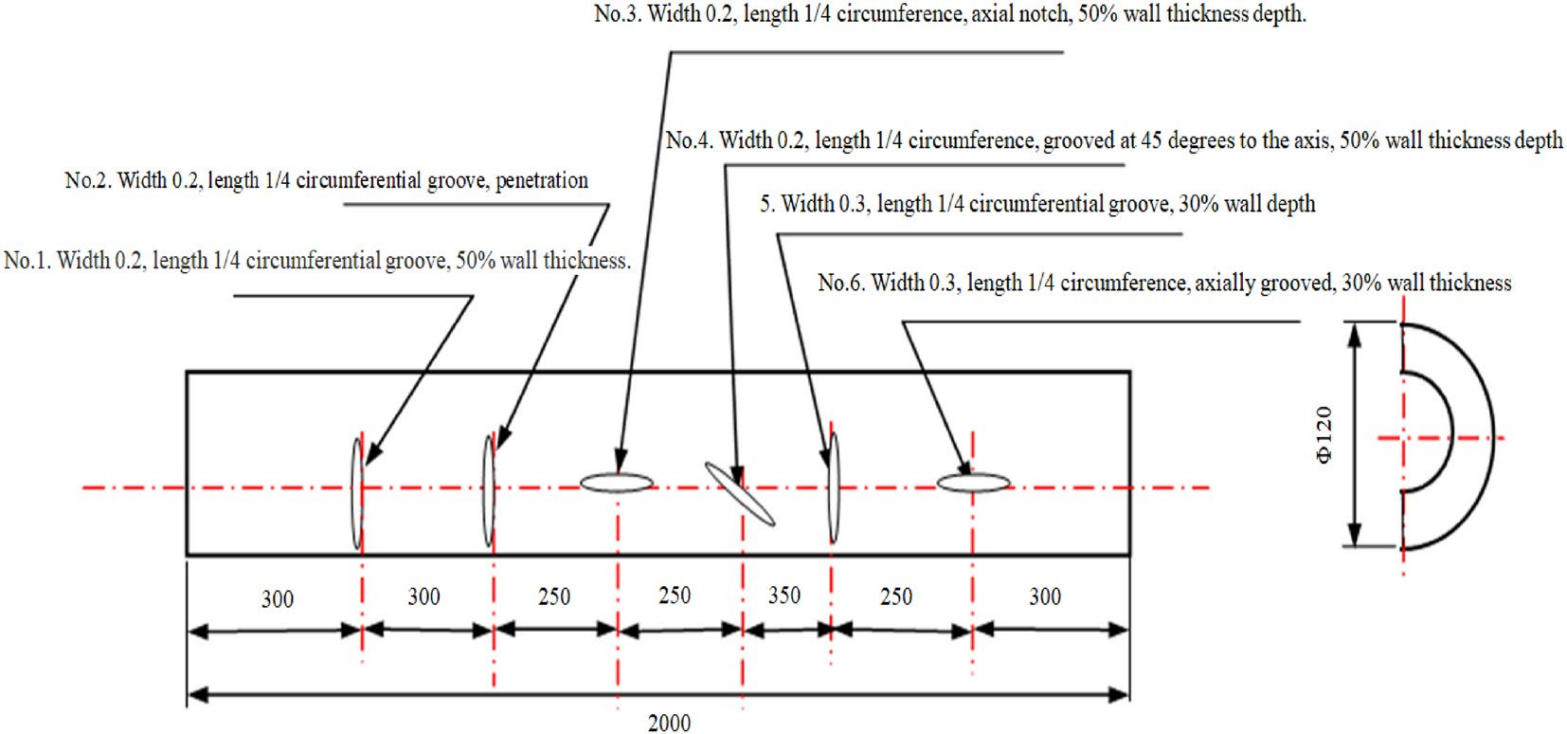
Dimensions of cracking-type defects in simulated cracking furnace tubes (Unit: mm, Wall thickness: 10 mm).

Figure 4 illustrates the dimensions of the artificial prefabricated simulated crack defects. These defects were designed to simulate the cracks in furnace tubes during actual usage, resulting from creep and carburization. Six groove-like defects with different angles and depths in the axial direction were intentionally created to mimic the irregularity of cracks observed in real furnace tubes. In which defects 1 and 2 were positioned 90 degrees apart in the circumferential direction, with Defect 3 between them. Defects 4 and 1 formed a 180-degree angle in the circumferential direction, while Defects 5 and 6 were separated by 180 degrees circumferentially.This setup allows for assessing the effectiveness of ultrasonic guided waves and low-frequency electromagnetic detection for cracks in various directions.

**Figure 5:**
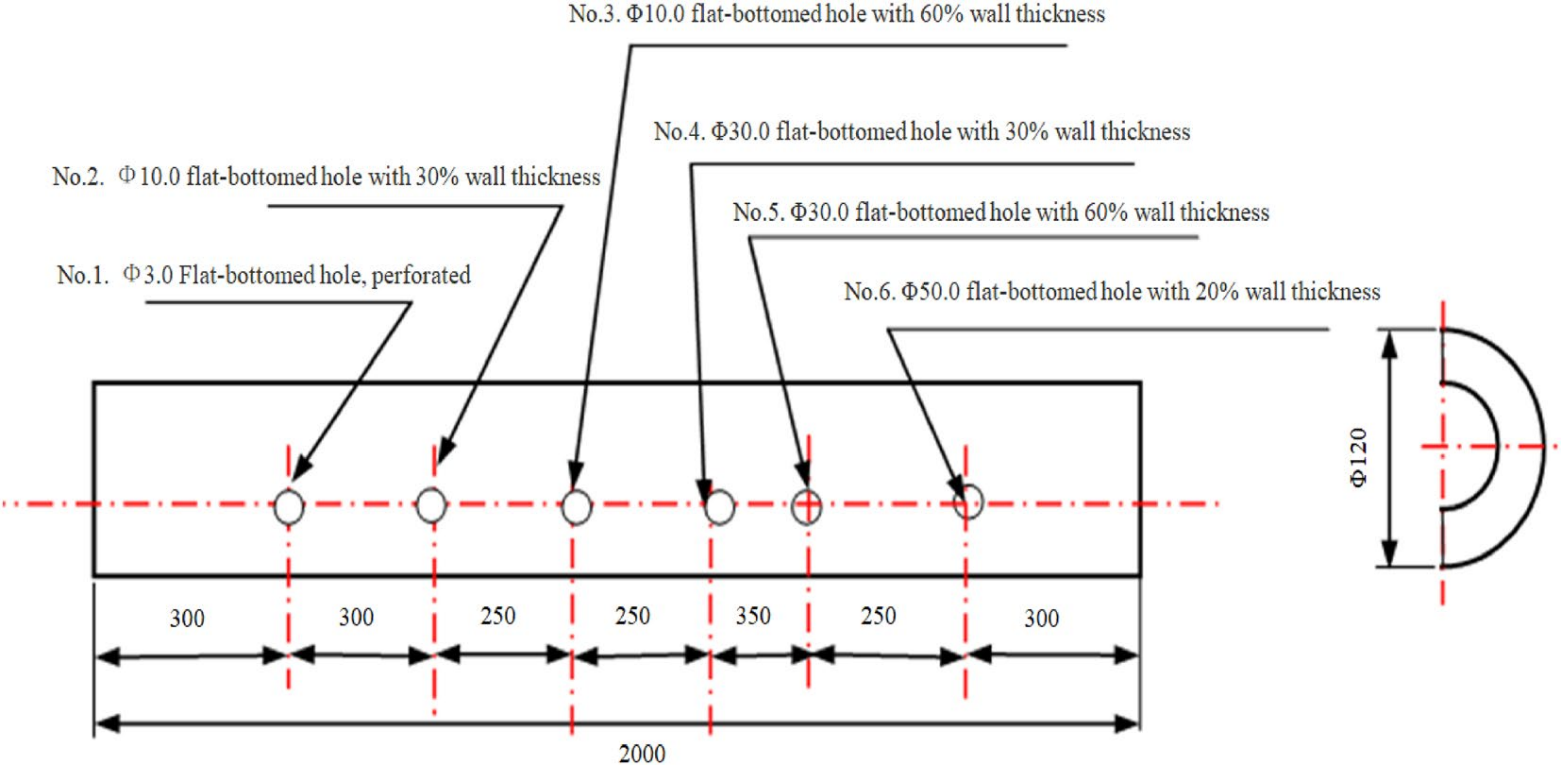
Dimensions of corrosion-type defects in simulated cracker tube specimens (Unit: mm, Wall thickness: 10 mm).

Figure 5 depicts the dimensions of the artificial prefabricated simulated corrosion defects. These defects primarily represent corrosion thinning in furnace tubes caused by high-temperature sulfur corrosion, high-temperature oxidation, or erosion. At the same time, these defects are also set at different angles, defects 2 and 1 were positioned at a 45-degree angle in the circumferential direction, and defects 3 and 2 were at a 45-degree angle in the circumferential direction. defects 4 and 3 formed a 45-degree angle in the circumferential direction, while defects 5 and 4 were positioned at a 45-degree angle in the circumferential direction. defects 6 and 5 were also at a 45-degree angle in the circumferential direction. This arrangement is helpful in verifying the effectiveness of the detection method for defect detection in different directions.

### Detection instruments and principles

#### Ultrasonic guided wave inspection instruments and principles

Magnetostrictive ultrasonic guided wave inspection is a widely used technique for inspecting and monitoring plates and pipes^19–21^. In this furnace pipe inspection experiment, we employed the magnetostrictive ultrasonic guided wave detector model MsSR 3030R. The instrument operated based on the principle that when a ferromagnetic material is subjected to an applied magnetic field, the magnetic field variation induces a small change in the material's physical length and volume, resulting in a magnetostrictive effect. By utilizing this effect within an alternating magnetic field, the detector generated elastic mechanical waves through repeated stretching and shortening. These waves propagated through the furnace tube, and when they encountered defects (sections with changes in cross-section), a portion of the waves was reflected and recorded by the detector. Analysis of the recorded signals provided corresponding test results.

#### Low-frequency electromagnetic detection instruments and principles

**Figure 6:**
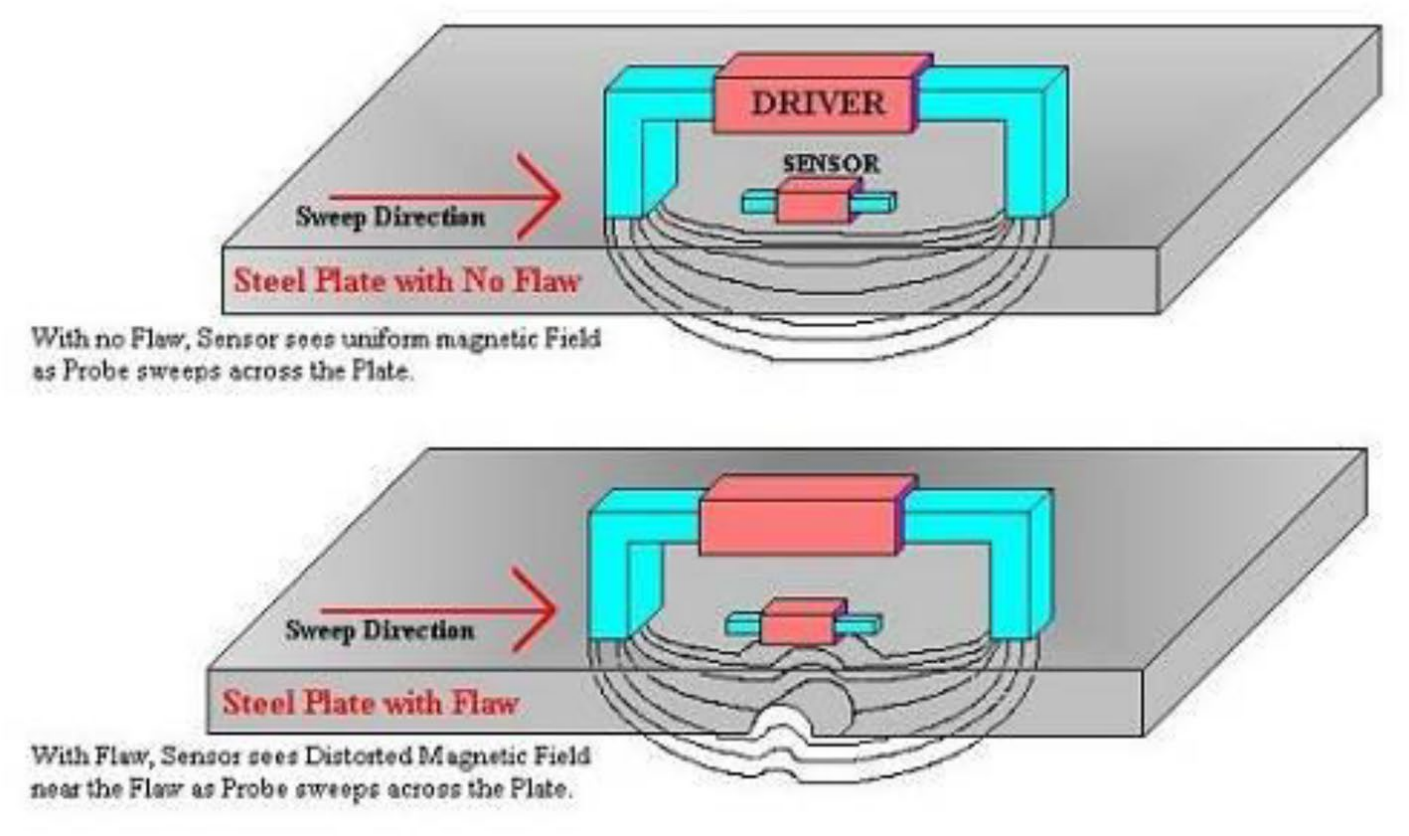
Schematic diagram of the detection principle of low-frequency electromagnetic technology.

Low-frequency electromagnetic detection technology is another widely employed method for pipeline inspection^22–24^. In this experiment, we utilized the TS 2000 low-frequency electromagnetic multi-channel furnace tube nondestructive testing system. This system was suitable for scanning and testing any pipe section from the outside and could detect surface and internal defects in both iron and non-iron materials while assessing the relative size of the defects. The system employed discrete excitation and reception sensor coils for low-frequency electromagnetic techniques. The excitation coil generated an alternating electromagnetic field at a low frequency (≤ 10 Hz), penetrating the measured material and transmitting from one side to the other. The electromagnetic field exhibited a certain signal intensity attenuation in areas without wall thickness reduction or defects. However, the probe detected a strong electromagnetic field signal when it encountered regions with wall thickness reduction or defects. Figure 6 illustrates a schematic diagram of the detection principle of low-frequency electromagnetic technology.

## Non-destructive testing results

### Ultrasonic guided wave detection results

Different adapters with central frequencies of 32 kHz, 64 kHz, and 128 kHz were selected, and the samples were tested to determine the most suitable adapter. In theory, higher detection frequencies result in higher detection accuracy.

#### Experimental results of specimens with simulated crack defects

**Figure 7:**
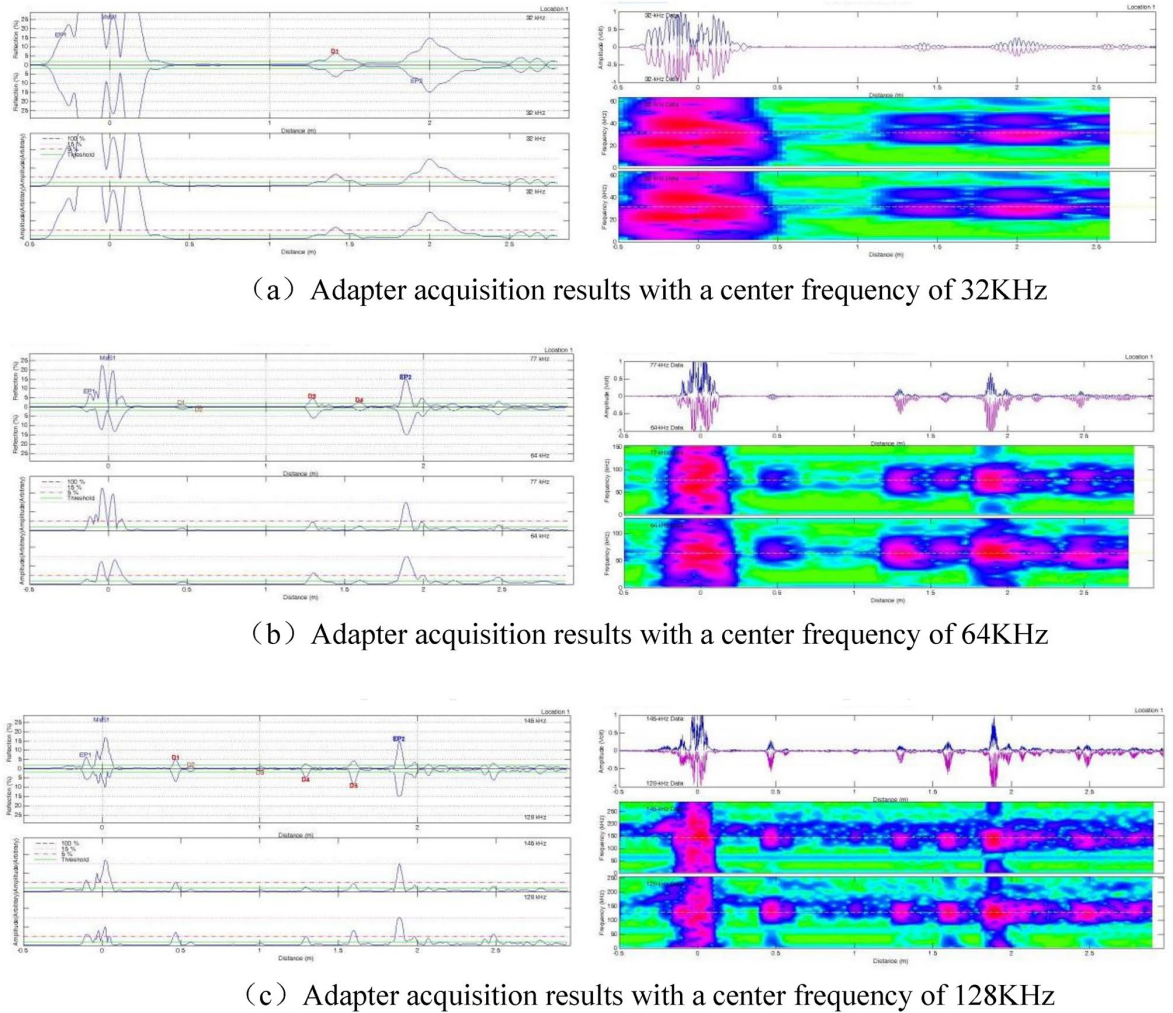
Adapter acquisition results for different center frequencies (distance vs. wave amplitude plots and corresponding spectrograms).

The specimens with simulated crack defects were tested using three adapters with different center frequencies: 32 kHz, 64 kHz, and 128 kHz. The detection results, including distance-amplitude diagrams and spectrograms, were obtained using the end of the pipe where defect 1 was the starting point. The results are presented in Fig. 7.

A comparison between Figs. 4 and 7 revealed that the adapter with a center frequency of 32 kHz could detect only one defect, while the adapter with a center frequency of 64 kHz could detect four defects. The adapter with a center frequency of 128 kHz exhibited the highest detection accuracy, allowing the detection of five defects. Therefore, the adapter with a center frequency of 128 kHz was chosen for subsequent tests.

**Figure 8:**
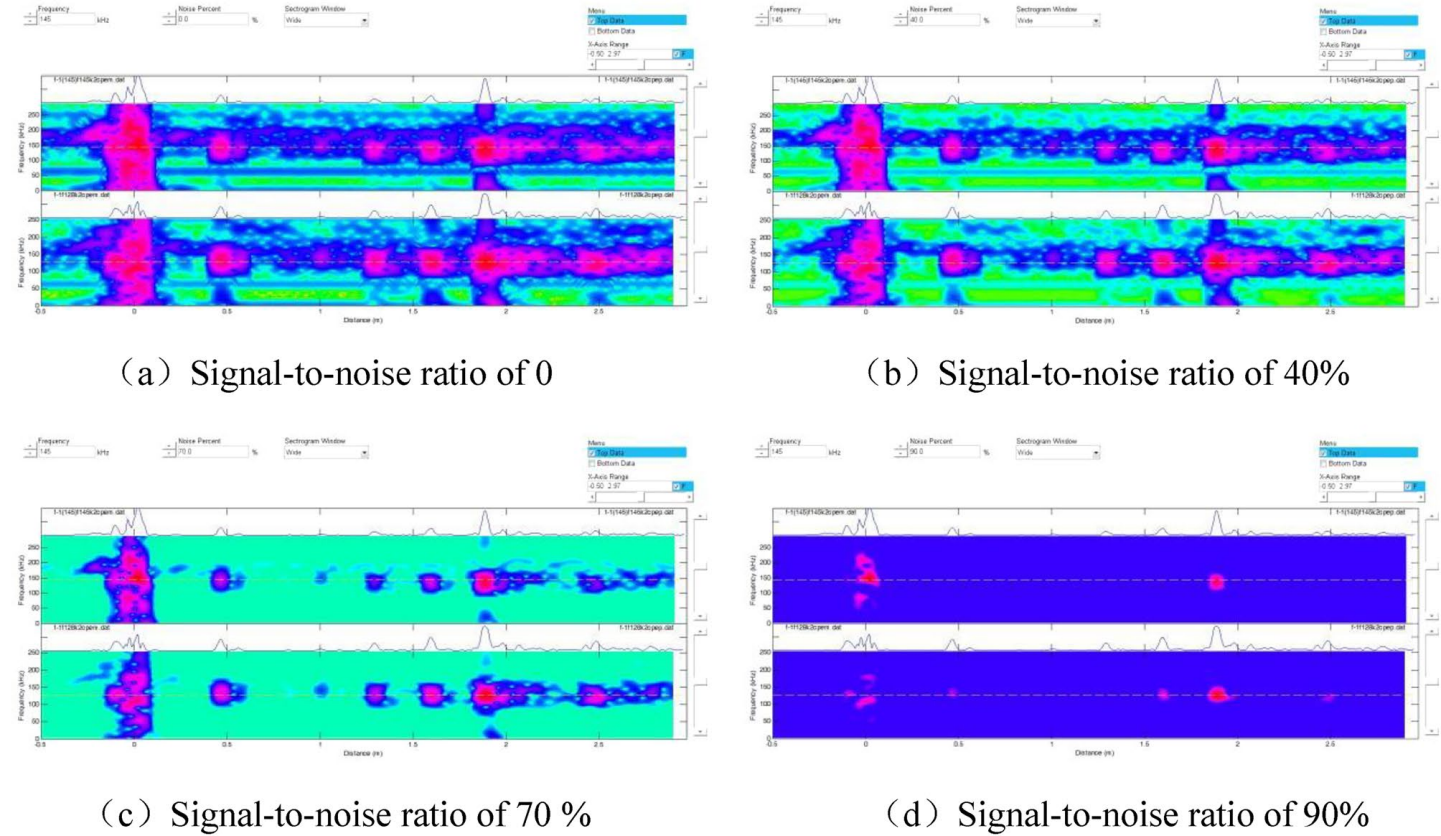
Spectrograms at different signal-to-noise ratios.

Unlike in Fig. 4, Defect 1 was not detected using the adapter with a center frequency of 128 kHz. By contrast, when the pipe end where Defect 1 was located was considered the starting end, and the signal-to-noise ratio was adjusted to analyze changes in the spectrogram. The results are shown in Fig. 8.

Figure 8 displays the signals corresponding to different defects on the spectrum. Since Defect No.1 was located only 200 mm from the initial end, the proximity caused the detection signal to be submerged by the initial wave signal, making it undetectable in the previous tests. Additionally, we noticed that each signal's amplitude differed, as indicated by the color on the spectrum. Hence, the detection rate of magnetostrictive ultrasonic-guided wave detection varies for crack-like defects in different orientations.

**Figure 9:**
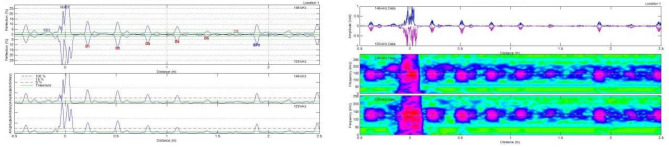
Detection results from the end of the tube where defect No. 6 is located using an adapter with a center frequency of 128 kHz.

To detect Defect No.1, we continued using the adapter with a center frequency of 128 kHz. However, we changed the starting point to the end of the pipe where Defect No.6 was located and repeated the test from a different direction. The results are shown in Fig. 9.

As illustrated in Fig. 9, all six defects were effectively detected. However, signals for Defects No.1 and No.2 in Fig. 4 were below the gate line, indicating that the orientation of the defects affects the signal amplitude.

The above test confirms that when ultrasonic guided waves are used to detect cracks in the furnace tube, the orientation of the crack defects does not affect the signal detection, but it does cause a decrease in signal amplitude.

**Figure 10:**
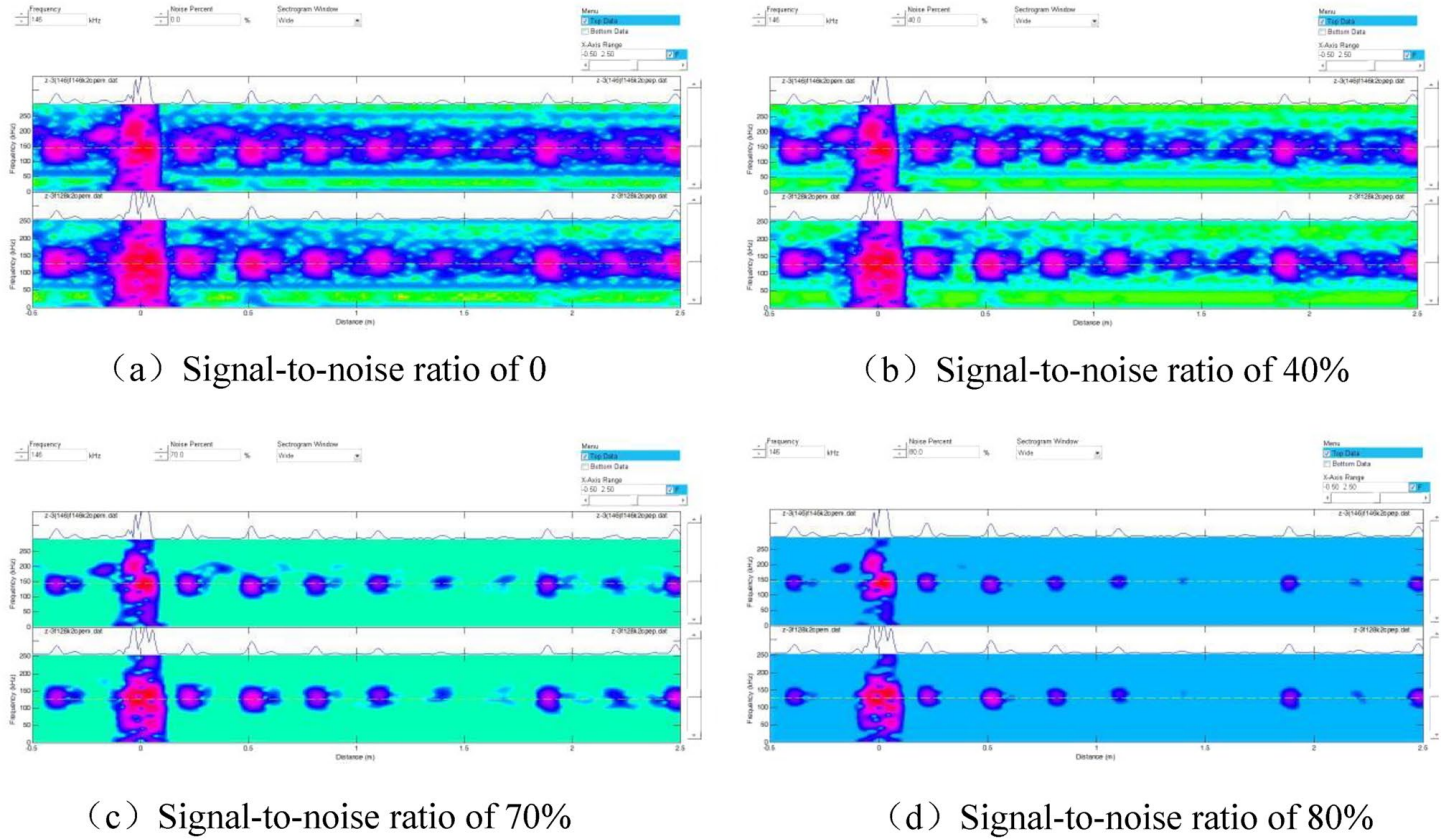
Spectrograms at different signal-to-noise ratios.

The signal-to-noise ratio was further adjusted to observe the defect distribution on the specimen and identify the source of suspicious signals, as depicted in Fig. 10.

As illustrated in Fig. 10a–c, the signals corresponding to the six prefabricated defects were observed, and the defect signals became stronger with the increase in the signal-to-noise ratio. However, when the signal-to-noise ratio reached 80% (Fig. 10d), smaller defect signals were easily filtered out, decreasing the detection rate.

#### Test results of specimens simulating corrosion-like defects

**Figure 11:**
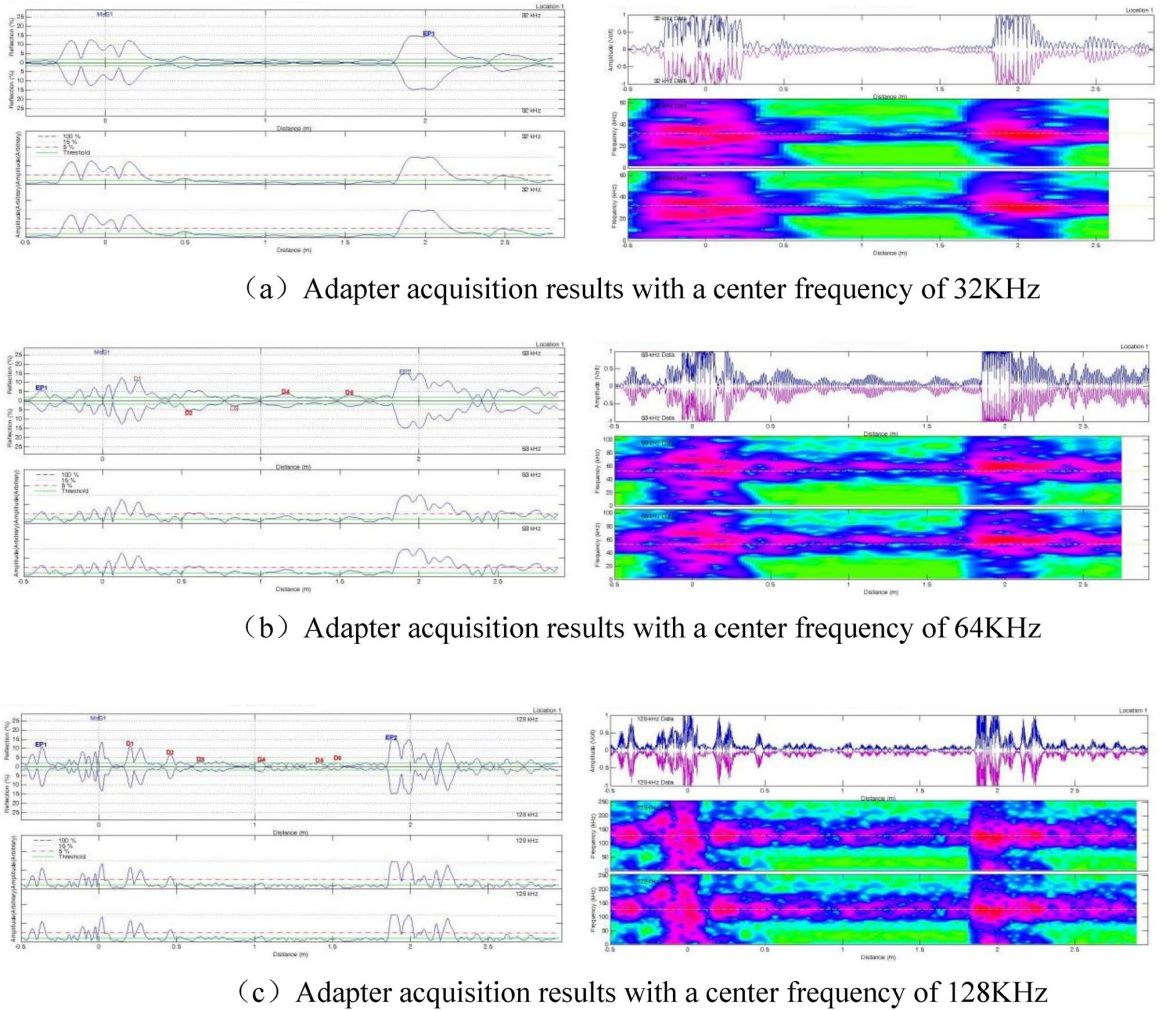
Adapter acquisition results for different center frequencies (distance-wave amplitude plots and corresponding spectrograms).

Similar to the previous test, we used three adapters with center frequencies of 32 kHz, 64 kHz, and 128 kHz. The specimen with simulated corrosion defects was tested using the end of the pipe where Defect No.6 was located as the starting point. The results are presented in Fig. 11.

As illustrated in Fig. 11, the adapter with a center frequency of 128 kHz exhibited the highest detection accuracy for corrosion defects and could detect all six defects simulated in the specimen. Subsequent experiments utilized the 128 kHz adapter.

**Figure 12:**
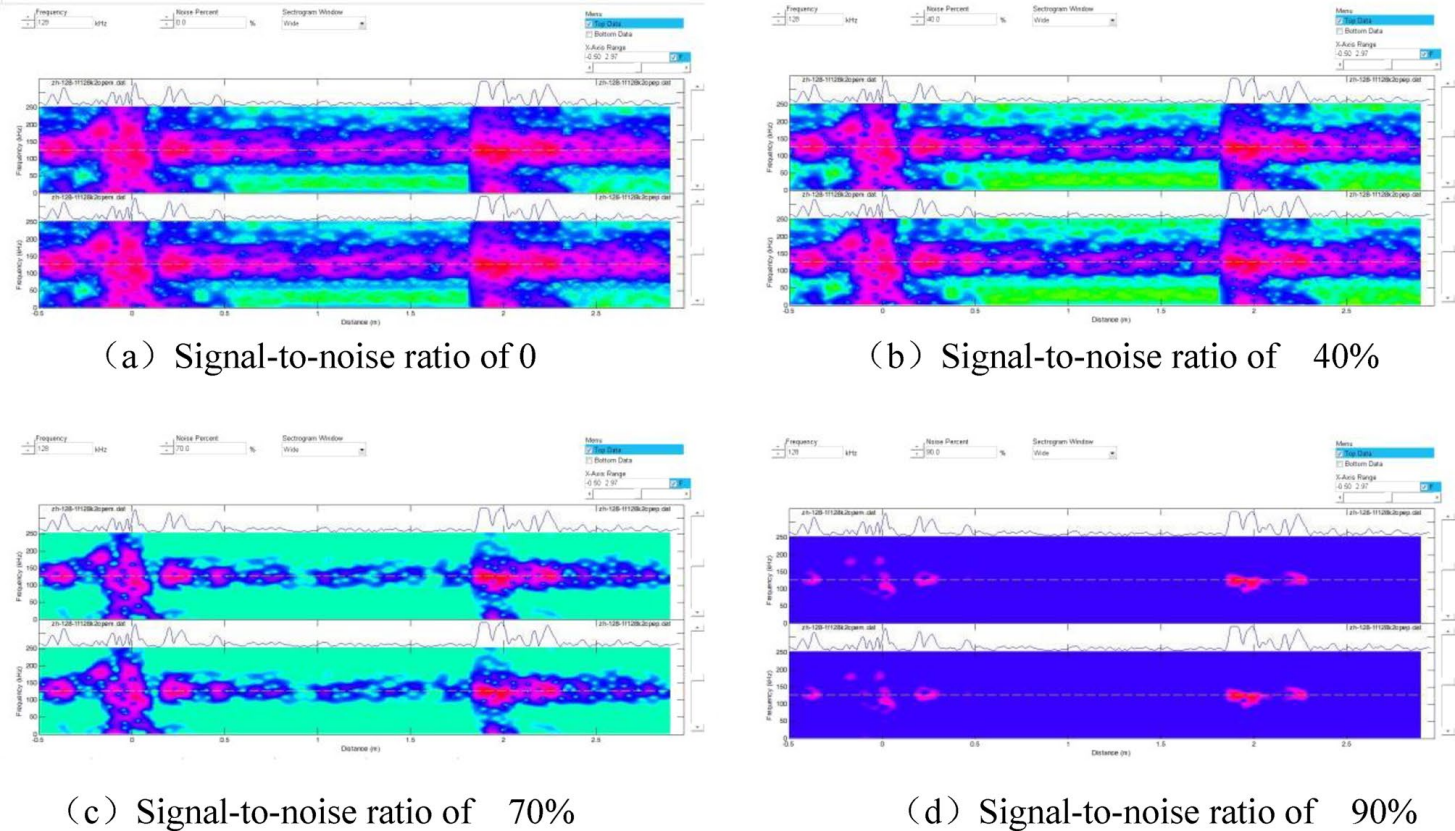
Spectrograms at different signal-to-noise ratios.

By adjusting the signal-to-noise ratio, we observed changes in the spectrogram, as shown in Fig. 12.

As illustrated in Figs. 11 and 12, magnetostrictive ultrasonic guided waves also effectively detect corrosion defects. However, a comparison of the spectra of crack defects revealed that the signal amplitude for corrosion defects was smaller, resulting in weaker colors on the spectra. Thus, ultrasonic guided waves are more effective in detecting crack defects in cracking furnace tubes than corrosion defects.

### Low-frequency electromagnetic detection results

The same set of test objects used in the ultrasonic guided wave testing, namely cracking furnace tubes with crack-like defects and flat-bottomed hole defects, were employed for low-frequency electromagnetic detection. The defect sizes are shown in Figs. 4 and 5.

#### Experimental results of specimens with simulated crack defects

**Figure 13:**
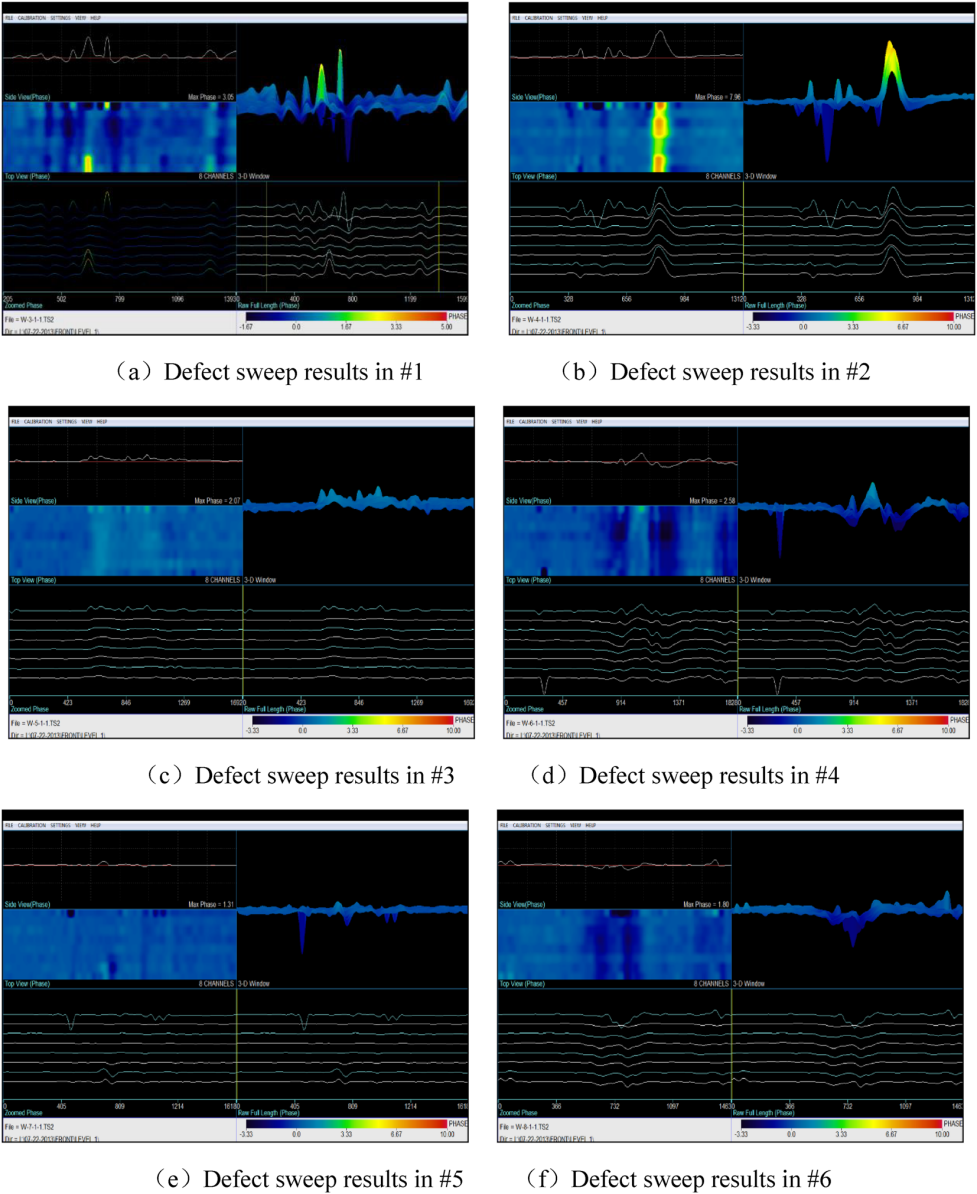
Detection results of 6 crack-like defects.

Figure 4 illustrates the presence of six crack-like defects on the furnace tube. The scanner was calibrated on a defect-free part of the furnace tube, with the defect-free part as the reference signal. The defects were labeled as No.1–No.6 in the order from left to right in Fig. 4 and then scanned using low-frequency electromagnetic detection. The experimental data are presented in Fig. 13.

The detection results shown in Fig. 13 indicated that low-frequency electromagnetic technology accurately detected the crack defects in the furnace tube. The maximum phase amplitude values of the defect signals differed based on the size of the defects and their orientations. For instance, comparing (a), (b), and (e) in Fig. 13 revealed that the signal intensity varied with different defect depths. Defect No.1, with a depth equal to 50% of the wall thickness, exhibited a phase amplitude of 3.06. Defect No.2, with a depth equal to 100% of the wall thickness, exhibited a phase amplitude of 7.98. Defect No.5, with a depth equal to 30% of the wall thickness, exhibited a phase amplitude 1.31, indicating that larger defect sizes result in higher signal intensities. Additionally, a comparison between (a), (c), and (d) in Fig. 13 revealed that defects of the same size produced different maximum phase values because of their orientations. Defects perpendicular to the magnetic lines of force exhibited larger phase values, while defects parallel to the magnetic lines exhibited smaller values 3.06, 2.07, and 2.58.

#### Experimental results of specimens with simulated corrosion defects

**Figure 14:**
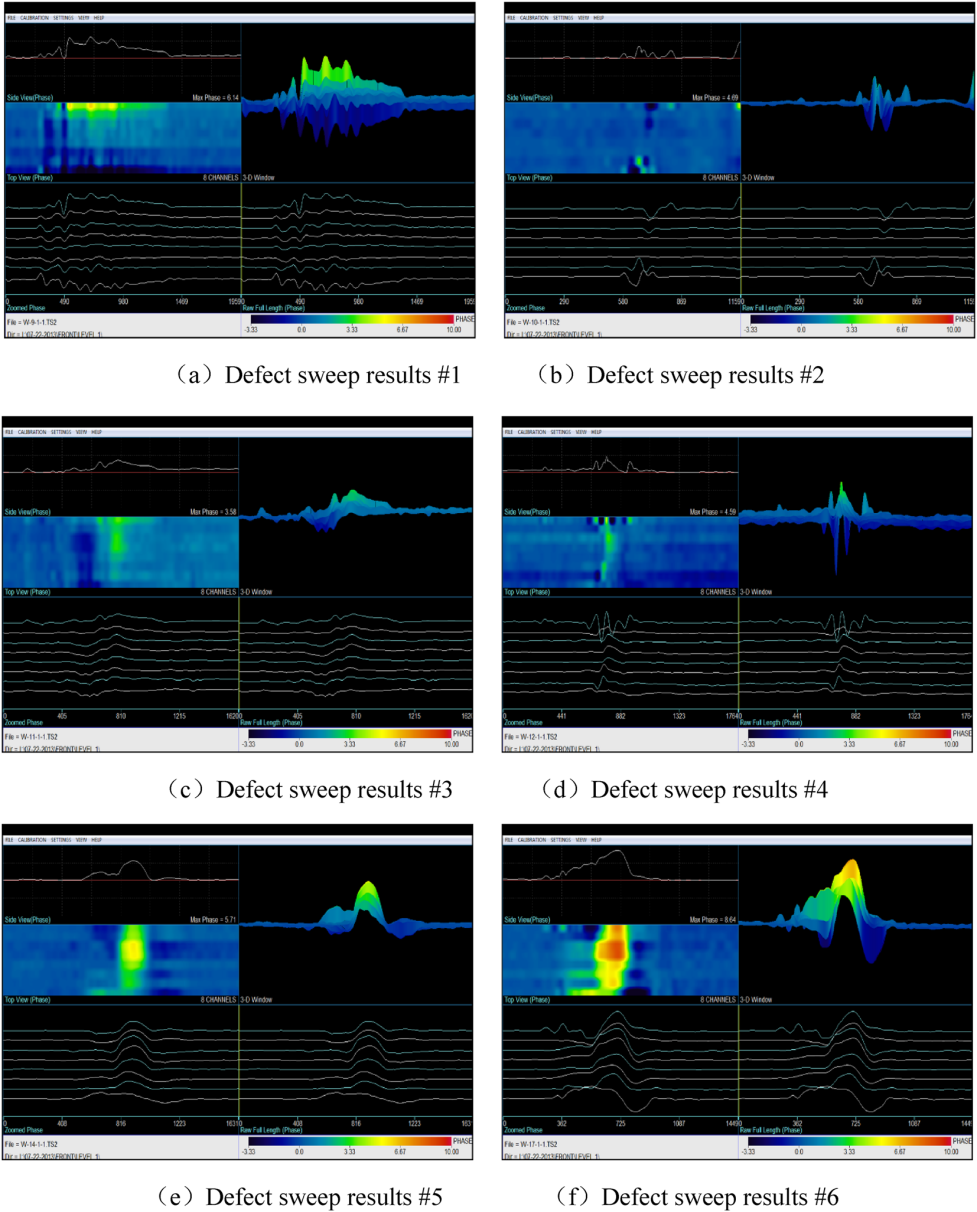
Detection of 6 flat-bottomed porous corrosion defects.

Figure 5 displays the presence of six flat-bottomed hole-like defects on the furnace tube. Similar to the previous test, the scanner was calibrated on a defect-free part of the furnace tube, with the signal from the defect-free part serving as the reference. The defects were labeled as No.1 to No.6 in the order from left to right in Fig. 5 and then scanned using low-frequency electromagnetic detection. The experimental data are presented in Fig. 14.

The detection results indicated that low-frequency electromagnetic technology accurately detected the simulated corrosion defects in the form of flat-bottomed holes. The maximum phase values obtained from the detection of defects increased with the defect size. Therefore, after calibrating the instrument on the sample tube, the size of defects could be determined based on the maximum phase amplitude of the signal.

## Discussion

### Ultrasonic-guided wave detection

Multiple tests were conducted on two groups of specimens with crack-like and corrosion-like defects using different center frequency adapters and signal-to-noise ratios. The results demonstrated that the best detection effect was achieved using an adapter with a center frequency of 128 kHz. As the signal-to-noise ratio increased, the defect signal became stronger. However, excessively high signal-to-noise ratios could filter out smaller defect signals, decreasing the detection rate. Therefore, during on-site inspections, it was necessary to adjust the signal-to-noise ratio and select the most suitable ratio to identify more suspicious defect signals and enhance the effectiveness of detection. Magnetostrictive ultrasonic-guided wave detection exhibited directionality in detecting crack defects. The detection rate was higher for cracks perpendicular to the axial direction of the furnace tube compared to cracks parallel to the axial direction.

The weak signal amplitude and spectral color observed for corrosion defects in the experiment confirmed that the prefabricated corrosion defects in the specimen were processed as flat-bottomed holes. The arc-shaped interface generated by the flat-bottomed hole diffracted the front edge of the detection signal, leading to a decrease in the detection signal amplitude. However, actual corrosion defects in cracking furnace tubes are irregular, not regular flat-bottomed holes. Therefore, the detection signal intensity for actual corrosion defects would be stronger than that simulated in this experiment, indicating that the ultrasonic-guided wave detection method can also be applied to corrosion defects.

### Low-frequency electromagnetic detection

The results of low-frequency electromagnetic detection indicated its effectiveness in detecting crack and corrosion defects in furnace tubes. After calibrating the sample tubes, the size and position of defects could be quantitatively analyzed. Comparing Figs. 13 and 14 revealed that the maximum phase amplitude of detection results for flat-bottomed hole defects was generally larger than for cracks. This difference may be attributed to the larger volume loss of flat-bottomed hole defects, resulting in different phase values for defects.

Several factors should be considered when conducting detection using low-frequency electromagnetic technology:

1. Calibration of the reference signal on the sample tube was essential, similar to other electromagnetic detection technologies such as eddy current detection. Calibration using different defects allowed for defect size determination, providing a basis for defect analysis.
2. Most cracks in furnace tubes originate from one-third of the inner surface. Because of the directional nature of columnar crystal faces, most cracks extended radially along the grain boundaries. Therefore, comparative test data on artificial radial cracks were more meaningful.
3. The detection speed during the process is crucial. In addition, comparing Figs. 12 and 13 revealed that the waveform of flat-bottomed hole defects was larger than that of crack defects. After calibration on the calibration tube, the furnace tube could be detected at a certain speed, and the nature of defects could be determined based on the waveform. Excessive scanning speed or an imbalanced scanning process could hinder defect analysis and result in missed inspections.

### Combination of ultrasonic guided wave and low-frequency electromagnetic for cracker furnace tube detection

The experimental investigation of the two detection methods revealed their advantages in detecting cracking furnace tube defects. Ultrasonic-guided wave detection offered high detection speed and rapid defect localization, making it suitable for large-scale screening of cracking furnace tube defects. Although low-frequency electromagnetic detection has a lower scanning speed than ultrasonic-guided wave detection, it can be used to qualitatively and quantitatively assess the defective or suspected parts. Moreover, low-frequency wave-detection can be combined with calculations of strength and residual life to effectively determine the continuous usability of cracking furnace tubes. Therefore, a new inspection and detection method for cracking furnace tube defects was developed based on the experimental results. This method combines ultrasonic-guided wave detection with low-frequency electromagnetic detection for rapid defect localization and accurate quantitative evaluation of cracking furnace tube defects.

## Conclusions

During high-temperature service, Cr35Ni45Nb cracking furnace tubes undergo material deterioration and produce defects. However, conventional single inspection methods are deficient in the effectiveness and convenience of cracker tube inspection. In this study, a combination of ultrasonic guided wave and low-frequency electromagnetic detection is proposed for the first time to improve the efficiency and accuracy of cracker tube defect detection.

An experimental study of ultrasonic guided wave detection and low-frequency electromagnetic detection was carried out by prefabricating cracks and corrosion defects of different orientations and sizes on cracking furnace tubes that had been in service for 2 years. The results show that ultrasonic guided waves can realize rapid screening of defects when adjusted to the appropriate center frequency adapter and signal-to-noise ratio, and that the low-frequency electromagnetic detection technique is capable of qualitatively and quantitatively analyzing defects by calibrating the instrument on a sample tube with standard defects. Therefore, based on the experimental results, a new inspection method combining the two inspection modes was innovatively developed, which can be used for rapid defect localization and accurate quantitative evaluation of cracker furnace tubes. In addition, the experimental study is based on the in-service furnace tube whose structure has deteriorated after two years of service, and its experimental results show its applicability and effectiveness in practical engineering application.This method is of great significance in practical engineering applications and can create great economic value.

Future studies will examine a broader selection of tubes and conduct additional laboratory investigations and on-site inspection tests to facilitate the early implementation of the proposed technology for detecting cracker tube defects, thereby enhancing the long-term safety of cracker furnaces.

## Data Availability

The datasets used and/or analysed during the current study available from the corresponding author on reasonable request.
